# Contribution to the improvement of the nutritional and functional properties of bread by incorporating cinnamon powder (*Cinnamomum verum*)

**DOI:** 10.1002/fsn3.3564

**Published:** 2023-07-13

**Authors:** Koffi Maïzan Jean‐Paul Bouatenin, Fatoumata Camara, Yabo Majoie Geroxie Tohoyessou, Wahauwouélé Hermann Coulibaly, Zamblé Bi Irié Abel Boli, Gniré Abibata Ouattara, Marina Koussemon

**Affiliations:** ^1^ Department of Food Sciences and Technology, Laboratory of Biotechnology and Food Microbiology Nangui Abrogoua University Abidjan Côte d'Ivoire; ^2^ Department of Food Sciences and Technology, Laboratory of Nutrition and Food Safety Nangui Abrogoua University Abidjan Côte d'Ivoire; ^3^ Biochemistry and Cell Biology Department, Faculty of Sciences and Techniques Biology and Molecular, Typing in Microbiology Laboratory University of Abomey‐Calavi (FSS/UAC) Cotonou Benin

**Keywords:** bread, cinnamon, incorporation, nutritional and functional properties

## Abstract

Bread is a staple food for billions of households around the world; yet, some of its nutritional value is reduced during the manufacturing process. With this in mind, this work was carried out with the objective of improving the nutritional and functional properties of white bread by using cinnamon in breadmaking in order to contribute to the prevention of certain diseases related to eating habits. Therefore, bread‐making trials by incorporating 0%, 0.5%, and 1% of cinnamon powder were carried out. From then on, the breads produced underwent physicochemical, biochemical, and organoleptic analyses. Thus, the results showed that the biochemical composition of the bread containing 1% cinnamon powder and the bread containing 0.5% cinnamon was 11.96 ± 0.02% and 11.22 ± 0.02% for protein, 1.70 ± 0.01% and 1.41 ± 0.07% for fiber, respectively, compared to 10.76 ± 0.014% protein and 1.36 ± 0.17% fiber for the bread without cinnamon. In terms of phytochemical composition, the bread containing 1% cinnamon powder recorded the highest contents of polyphenols, flavonoids, and condensed tannins (551.295 ± 25 μg EAG/g DM, respectively; 53.117 ± 1.36 μg EQ/g DM and 269.837 ± 39.2 μg EC/g DM) compared to the bread containing 0.5% of cinnamon powder and the bread with 1% cinnamon. From the nutritional and phytochemical point of view, the results of this work showed the positive impact of the incorporation of cinnamon powder in wheat flour‐based bread with beneficial properties on the health of consumers.

## Graphical abstract

This study has demonstrated the influence of the incorporation of 0.5% and 1% of cinnamon powder in bread. From the organoleptic point of view, the bread with 0.5% cinnamon was more appreciated by the panelists than the bread with 1% cinnamon. Cinnamon has, therefore, a good nutritional potential to be used in the food industry and to develop new products.

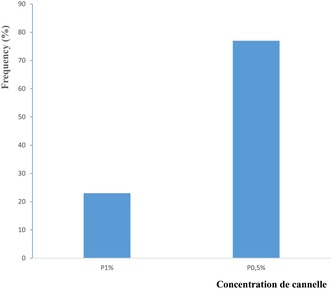

## 1 INTRODUCTION

The basis of the human diet is cereals and root and tuber crops. Due to their starch and protein contents, cereal foods are the most important source of energy in our diet and their protein fraction provides a wide range of amino acids (Della Valle et al., 2019). In addition, the recommendations of the National Nutrition and Health Program (PNNS) have emphasized the consumption of complex carbohydrates in general and bread in particular (PNNS 1: 2001–2005; PNNS 2: 2006–2010). This place that the PNNS dedicates to the consumption of bread takes all its sense in Côte d'Ivoire. Consumed in a common way, fermented product resulting from the flour of wheat, the bread became the daily food of the Ivorians and the basic food of billion households in the world. Rich in carbohydrates, bread is now consumed by 80% of the Ivorian population, making it the second most popular food after attiéké (Abiyou, 2021). The industrialization of food has mainly emphasized the presence of energy nutrients such as carbohydrates, proteins, and fats in our diet, to the detriment of the nonenergy fraction consisting of fiber, minerals, and other micronutrients. There is, therefore, an abundance of energy macronutrients, but they do not cover the recommended intake of minerals and micronutrients (Leenhardt, 2005). This is the case for bread, which is increasingly produced using refined flours, some of whose nutritional value is reduced during the manufacturing process (Rémésy et al., 2015). Since bread is an everyday food, it is important to understand the impact of its consumption on health at the lifetime level (Rémésy et al., 2015); hence, the need to develop new techniques improve its nutritional and functional values. The improvement of these nutritional and functional values of bread can be done at the level of the raw material, milling processes, or bread‐making methods (Rémésy et al., 2007). In the same vein, the use of herbs and spices has long served to improve cordial and tonic remedies. They were also used to preserve foods through their antifungal properties and to add odor, aroma, and flavor to foods. Spices and herbs can be rich sources of antioxidants, hence, the need to introduce them into consumer food products in order to make them functional and provide benefits to the consumer (Belloul & Zourane, 2020). Such is the case of cinnamon (*Cinnamomum verum*), a spice in the form of bark, a source of natural antioxidants due to its phytochemical composition in phenolic compounds. Also, rich in carbohydrates, essential minerals, and especially in dietary fiber, it seems that cinnamon is a preferred ingredient in our dishes. Moreover, apart from its aroma and its unique virtues, it has been demonstrated that even if the mechanisms involved in the absorption of cinnamon components by the body remain unknown, its beneficial action on oxidative stress is recognized (Prior & Gu, 2005; Shan & Cai, 2005). Thus, its use in breadmaking could contribute to the well‐being of consumers, especially those from vulnerable groups with limited access to proper health care. This is to prevent or preserve health by providing the body with an adequate supply of nutrients and micronutrients while avoiding excesses (Rémésy et al., 2015). In the literature, several researches have been done on cinnamon (*Cinnamomum verum*) but few researches have been done on the formulation of breads incorporated with cinnamon. Thus, the general objective of this study is to improve the nutritional and functional properties of white bread by using cinnamon in breadmaking in order to contribute to the prevention of some diseases related to eating habits.

## 2 MATERIALS AND METHODS

### 2.1 Material

The working materials are wheat flour type 55, cinnamon sticks, Eagle baker's yeast (*Saccharomyces cerevisiae*), and a bread improver IBIS Rouge from the international group Lesaffre.

### 2.2 Methods

#### 2.2.1 Realization of the bread‐making process

The process of breadmaking consists of a successive realization of the operations necessary to obtain bread on a laboratory scale. In order to find out the appropriate levels of cinnamon powder to be incorporated in cinnamon breads, preliminary studies were carried out in the laboratory by testing the fermentation of the dough with cinnamon powder levels of 0.5%, 1%, 2%, 10%, and 15%. An optimal fermentation of the dough was obtained in the cases of 0.5%, 1%, and 2% of cinnamon incorporated while in the other two rates, no fermentation was observed. However, an accentuated smell and taste of the cinnamon breads was noticed with the 2% rate, which is not the case with 0.5% and 1%. For this reason, the incorporation rates of cinnamon were set at 0.5% and 1% in the bread‐making tests. The bread‐making protocol was developed according to the data mentioned in Table 1. Thus, the breads are produced according to the technological diagram model of yeast breadmaking of De Tonnac (2010).

**TABLE 1:** Composition of control and experimental breads.

Ingredients	Control bread 100% wheat	Wheat bread + 0.5% cinnamon powder	Wheat bread + 1% cinnamon powder
Flour	100% = 5000 g	99.5% = 4975 g	99% = 4950 g
Cinnamon	0%	0.5% = 25 g	1% = 50 g
Water + Ice	3 L	2985 mL	2970 mL
Yeast	100 g	100 g	100 g
Improver	15 g	15 g	15 g
Salt	75 g	75 g	75 g

#### 2.2.2 Biochemical and phytochemical characteristics of the breads constituted

The percentage of dry matter (DM) and moisture content were determined gravimetrically in an oven at 105°C until a stable weight was obtained by Association of Official Agricultural Chemists (AOAC, 1990). The results were shown in percentage. Total ash content was determined by previous carbonization of the dry samples followed by incineration in an oven at 550°C (AOAC, 1990). The results were expressed in percentage. The extraction of the lipid fraction was carried out using a Soxhlet Tecator in accordance with AOAC (1990) method. The results were expressed in percentage. The total nitrogen determination was carried out using the Kjeldahl method (AOAC, 1990) and total protein was calculated by multiplying the total nitrogen by 6.25, the conversion factor calculated from the amino acid of total sample. The results were expressed in percentage. The total carbohydrate content was obtained by the difference of protein, moisture, and lipid. The Weende method described by Wolf (1968) was used for crude fiber determination. After extraction, the residue obtained was washed several times with hot water until complete removal of alkali and then oven‐dried at 105°C for 8 h, cooled in a desiccator, and weighed. After weighing, the residue was incinerated in an oven at 550°C for 3 h, cooled in a desiccator, and weighed again. The crude fiber content was expressed as percentage. The total energy value (TEV) was calculated using the traditional conversion factors for proteins (4 kcal/g), lipids (9 kcal/g), and carbohydrates (4 kcal/g) according to FAO (2006). The results were expressed in kcal/100 g of dry matter. The minerals were determined by atomic absorption spectrophotometry according to the AOAC (1990) digestion method using strong acids. The content of each mineral element was determined using a VARIAN AA.20 brand flame atomic spectrophotometer at a specific wavelength by comparison with standard solutions. Total polyphenol contents were determined using the colorimetric method of Folin–Ciocalteu (Singleton et al., 1999). The absorbance was read at 760 nm against a blank without extract taken as reference. Quantification of total polyphenols is done according to a linear calibration line (*y* = *ax* + *b*) performed by a standard extract gallic acid at different concentrations (0–1000 μg/mL). Results are expressed as microgram of gallic acid equivalent per gram of dry matter (μg GAE/g DM) of the powders. Total flavonoids assay was performed according to the method of Hariri et al. (1991) modified. The absorbance was read at 404 nm and compared to that of quercetol taken as standard (0.05 mg/mL). The percentage of total flavonoids is calculated as quercetol equivalent. The determination of condensed tannins in the different extracts was performed according to the method described by Heimler et al. (2006). To 400 μL of each sample or standard, 3 mL of a 4% methanolic vanillin solution and 1.5 mL of concentrated hydrochloric acid were added. The mixture is incubated for 15 min and the absorbance is read at 500 nm. Concentrations of condensed tannins are deduced from calibration ranges established with catechin (300 μg/mL) and are expressed as microgram of catechin equivalent per milligram of extract. For the determination of antioxidant activity by DPPH, the method used was that of Blois (1958) with slight modifications. DPPH is solubilized in absolute EtOH to obtain a solution with a concentration of 0.03 mg/mL. In dry and sterile tubes, 1 mL of extract solution to be analyzed, and 2 mL of DPPH solution is introduced. After shaking, the tubes are placed in the dark for 30 min. The absorbance of the mixture is measured at 517 nm against a blank formed by 2 mL DPPH solution +1 mL absolute EtOH.

#### 2.2.3 Realization of the tasting panel of the breads formulated

Organoleptic tests are tests allowing to evaluate the perceptions of a person toward a product, if he/she likes the product or not. Organoleptic tests are used by the food industry in the development of new products or the reformulation of existing products. Thus, in an environmental setup for tasting (a shed), the organoleptic test of cinnamon‐based breads was conducted by a panel of tasters consisting of 60 people. This number meets the requirements of the AFNOR XP V09‐501 standard which imposes a minimum of 60 individuals for a hedonic study, the CTIFL advises 90 individuals to obtain sufficient precision (Lespinasse et al., 2002). The objective of this test was to evaluate the acceptability of breads incorporated with 0.5% and 1% of cinnamon powder, respectively, by the tasters by appreciating the organoleptic characteristics of these breads compared to those of the baguette of bread they usually consume. For this purpose, each taster tasted separately the two breads in order to see which of the breads with 0.5% or 1% cinnamon powder was more satisfying, in order to determine their preferences for possible improvements of the organoleptic aspects of the formulated breads and their marketing for the general public consumption. Samples were evaluated by assigning them scores on a structured five‐level rating scale (from very unpleasant = 1 to very pleasant = 5), expressing the overall impression of their preference. The hedonic characteristics evaluated are as follows: color, taste, and smell. The overall acceptability of cinnamon breads was evaluated using a hedonic scale ranging from “unpleasant” to “pleasant” to “indifferent.” Organoleptic characteristics play an important role in determining the marketability of foods. The data collected from the tasting sheets were processed with Excel software to make radar diagrams.

#### 2.2.4 Statistical analysis

The software R.3.01, ANOVA method with post‐hoc test of Tukey, level of significance 5%, was used. This software allowed the calculation of the averages and the standard deviations of the physicochemical and biochemical parameters. It was also used to compare the averages of the parameters of the samples and to determine if the differences observed in the averages of the physicochemical, biochemical, phytochemical, and functional parameters were significant at the 5% level. AMP was performed with XLStat version 2016 software to group samples from all analyzed parameters.

## 3 RESULTS

### 3.1 Biochemical composition of the different breads formulated

The values of the different biochemical parameters of the breads produced from addition of cinnamon powder (cinnamon P) are presented in Table 2. Overall, a significant difference (*p* < .05) was observed between the bread samples regardless of the parameter analyzed. The bread enriched with 1% cinnamon P (1%) was characterized by higher concentrations of protein (11.96 ± 0.02%), iron (0.8 ± 0 g/kg), fat (0.27 ± 0%), dry matter (86.3 ± 0.14%), crude fiber (1.70 ± 0.01%), and a significant energy value of 337.1 ± 0.74 and a Ca:P ratio of 10.83 ± 0 lower than that of bread with 0.5% cinnamon (17.75 ± 0). On the other hand, the bread produced with 0.5% cinnamon P (0.5%) had high concentrations of phosphorus (0.049 ± 0 g/kg), calcium (0.87 ± 0 g/kg), magnesium (0.19 ± 0 g/kg), and ash (2.77 ± 0.07%). As for the control bread, it was rather characterized by a higher carbohydrate content of 72.59 ± 0.01% and an energy value higher (335.38 ± 0 kcal/100 g DM) than that of P (0.5%) (331.25 ± 0.42).

**TABLE 2:** Biochemical composition of the different formulated breads.

	P (0.5%)	P (1%)	P (0%)
Protein (%)	11.22 ± 0.02^a^	11.96 ± 0.02^a^	10.76 ± 0.01^c^
Phosphorus (g/kg)	0.049 ± 0^a^	0.048 ± 0^a^	0.035 ± 0^b^
Calcium (g/kg)	0.87 ± 0^a^	0.52 ± 0^b^	0.19 ± 0^c^
Iron (g/kg)	0.46 ± 0^a^	0.8 ± 0^b^	0.29 ± 0^c^
Magnesium (g/Kg)	0.19 ± 0^a^	0.15 ± 0^b^	0.13 ± 0^b^
Ash (%)	2.77 ± 0.07^a^	2.32 ± 0.06^a^	1.44 ± 0.01^b^
Dry matter (%)	85.25 ± 0.02^b,c^	86.3 ± 0.14^b^	84.76 ± 0.36^c^
Moisture (%)	14.75 ± 0.02^a^	13.7 ± 0.14^b^	14.99 ± 0.01^a^
Fat (%)	0.24 ± 0.12^a^	0.27 ± 0^a^	0.22 ± 0^a^
Crude fiber (%)	1.41 ± 0.07^a,b^	1.70 ± 0.014^a^	1.36 ± 0.17^b^
Ca:P	17.75 ± 0^a^	10.83 ± 0^b^	5.42 ± 0^c^
Carbohydrates (%)	72.51 ± 0.19^a^	70.24 ± 0.12^a^	72.59 ± 0.01^b^
Energy value (kcal/100 g DM)	331.25 ± 0.42^a^	337.10 ± 0.74^b^	335.38 ± 0^b^

*Note*: In the same line of each parameter, the values bearing the same letter do not show a significant difference at the 5% threshold.

Abbreviations: Ca:P, Calcium/Phosphorus ratio; DM, Dry matter; P (0%), control bread (bread without added cinnamon); P (0.5%), bread enriched with 0.5% cinnamon powder; P (1%), bread enriched with 1% cinnamon powder

### 3.2 Phytochemical characteristics of the breads formulated with cinnamon

**FIGURE 1:**
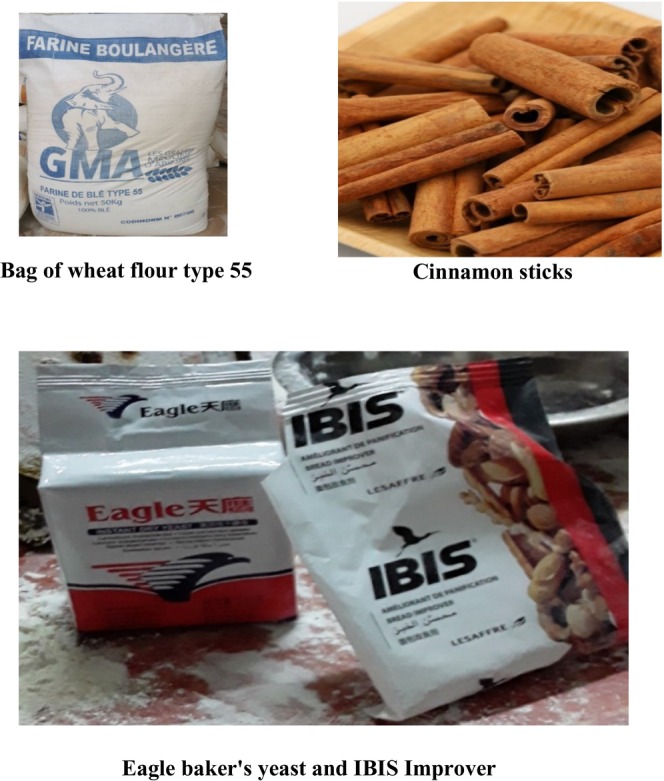
The working material.

The contents of phenolic compounds of the breads are presented in Table 3. The addition of cinnamon powder to the breads improved the total phenolic content. Thus, the content of total phenols increased from 292.345 ± 10.41 μg EAG/g DM in the bread without cinnamon addition (control bread) to 450.868 ± 64.13 μg EAG/g DM in the bread with 0.5% cinnamon addition (0.5% bread) and to 551.295 ± 25 μg EAG/g DM in the bread enriched with 1% cinnamon (1% bread). Also, statistical analyses showed a significant difference (*p* < .05) in total phenolic content between the breads. On the other hand, in terms of total flavonoids and condensed tannins, the effect of cinnamon addition was a function of the added rate. Thus, at 1% added cinnamon powder, the total flavonoid and condensed tannin contents increased from the control bread to the breads (1%) from 47.980 ± 3.75 to 53.117 ± 1.36 μg EQ/g DM and from 200.124 ± 46.99 to 269.837 ± 39.25 μg EC/g DM, respectively. In contrast, the addition of 0.5% cinnamon powder resulted in a decrease in total flavonoid and condensed tannin levels compared to the control bread. The levels decreased from 47.980 ± 3.75 to 40.534 ± 2.43 μg EQ/g DM for total flavonoids and from 200.124 ± 46.99 to 160.962 ± 14.88 μg EC/g DM for condensed tannins. However, a significant difference (*p* < .05) was observed between samples (Figure 1).

**TABLE 3:** Content of phenolic compounds in breads.

	Total phenols (μg EAG/g DM)	Total flavonoids (μg EQ/g DM)	Condensed Tannins (μg EC/g DM)
P (0%)	292.345 ± 10.41^a^	47.980 ± 3.75^a^	200.124 ± 46.99^a,b^
P (0.5%)	450.868 ± 64.13^b^	40.534 ± 2.43^b^	160.962 ± 14.88^b^
P (1%)	551.295 ± 25^c^	53.117 ± 1.36^a^	269.837 ± 39.25^a^

*Note*: In the same column of each parameter, the values bearing the same letter do not show a significant difference at the 5% threshold.

Abbreviations: DM, Dry matter; P (0%), control bread (bread without added cinnamon); P (0.5%), bread enriched with 0.5% cinnamon powder; P (1%), bread enriched with 1% cinnamon powder

### 3.3 Antioxidant activity of cinnamon‐based breads

**FIGURE 2:**
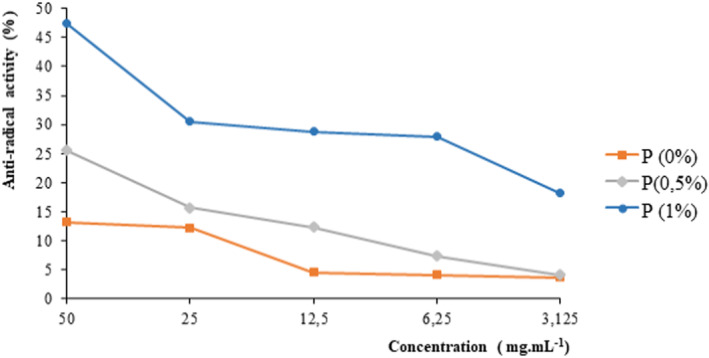
DPPH antioxidant profile of control bread (normal bread) and formulated breads. P (0.5%): bread enriched with 0.5% cinnamon powder, P (1%): bread enriched with 1% cinnamon powder, P (0%): control bread (bread without added cinnamon).

The antioxidant activity of breads, evaluated through the antiradical activity is presented in Figure 2. Regardless of the bread, the antiradical activity, the highest was recorded at 50 mg/mL. At the same concentration, the highest antiradical activity was observed in the bread with 1% cinnamon powder with 47.73 ± 0.01%. Then follows the bread enriched with 0.5% cinnamon powder with 25.59 ± 0.01% against 13.19 ± 0.02% for the control bread. Thus, a significant increase (*p* < .05) in the antiradical power is a function of the rate of the added cinnamon powder.

### 3.4 Results of the hedonic test of the different formulated breads

**FIGURE 3:**
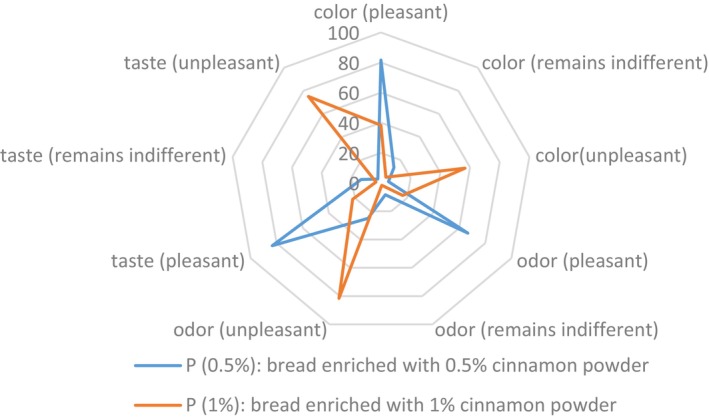
Organoleptic profile of cinnamon‐incorporated breads.

**FIGURE 4:**
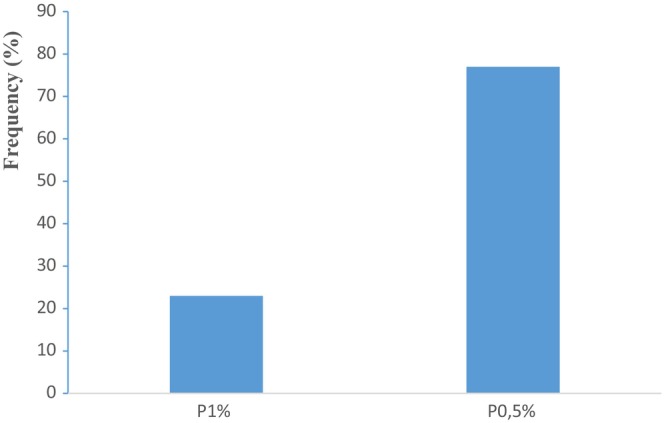
Acceptability rate of breads by panelists. P (0.5%): bread enriched with 0.5% cinnamon powder, P (1%): bread enriched with 1% cinnamon powder.

The data collected from the parameters such as color, taste, and smell of the produced breads were processed on Excel software for the realization of the radar diagram (Figure 3) and the histogram (Figure 4). The analysis of the figures shows that the panelists appreciate more the taste (83.33%), the color (81.67%), and the smell (66.67%) of the bread with 0.5% of cinnamon compared to the taste (21.67%), the color (38.33%), and the smell (16.67%) of the bread with 1% of cinnamon. Thus, respectively 75%, 56.67%, and 81.67% of panelists find unpleasant taste, color, and smell of bread with 1% cinnamon against, respectively, 3.33%, 5%, and 25% of tasters who find unpleasant taste, color, and smell of the bread with 0.5% cinnamon. Some of the panelists “remain indifferent” to the taste (13.34%), color (13.34%), and smell (8.33%) of bread with 0.5% cinnamon. As for the bread with 1% cinnamon, 3.33% remain indifferent to the taste, 5% to the color, and 1.66% to the smell.

## 4 DISCUSSION

The influence of the incorporation of cinnamon powder in bread can contribute to the improvement of its nutritional and functional properties. Thus, the evaluation of the biochemical parameters of the breads revealed that the fiber content increases with the incorporation of cinnamon powder, from 1.41 ± 0.07 for the bread with 0.5% cinnamon to 1.70 ± 0.014 for the bread with 1% cinnamon powder versus 1.36 ± 0.17 for the control bread. Indeed, increasing the dietary fiber content in cereal foods is considered a good way to reach the recommended fiber intake in adults (25–30 g per day (Lairon et al., 2005)). Also, the results also showed a protein content of 11.22 ± 0.02% for bread with 0.5% cinnamon powder and 11.96 ± 0.02% for bread with 1% cinnamon powder. These results are significantly higher than that of the control bread (10.76 ± 0.01%). In addition, the results revealed an increase in fat, fiber, ash, mineral, and energy content in the formulated breads except for the carbohydrate content which is higher in the control bread. This increase in fat, fiber, ash, and mineral content could be due to the enrichment of the bread with cinnamon and the reduction in carbohydrate content in the formulated breads could be explained by the increase in fat and protein content in these breads. These results are in agreement with those performed by Ouazib (2017) who states that the combination of wheat flour with legumes would decrease the amount of total carbohydrates in the final product due to the low amount of carbohydrates and the high amount of proteins and lipids in the legumes. The energy values of the formulated breads ranged from 331.25 ± 0.42 to 337.10 ± 0.74 kcal/100 g DM. These breads have a slightly higher energy value than the control bread and can, therefore, be recommended for the population's diet. In addition, increased consumption of mineral‐rich foods may improve mineral regulation and reduce the risk of cardiovascular disease and some cancers (Ismail et al., 2011). Globally, it is estimated that two billion people suffer from micronutrient malnutrition (Johns et al., 2006). Pregnant and lactating women and young children are the first victims of deficiencies, as their needs for vitamins and minerals are greater. They, therefore, suffer more from the harmful consequences of these deficiencies (Black et al., 2003). The majority of mineral and vitamin intake is, therefore, provided by fruits, vegetables, and cereals (Wargovich, 2000; Winston & Beck, 1999). Formulated breads seem to be an important source of minerals in the present study. Most of the highlighted minerals play an important role in the body. The analyses showed that the Ca/P ratio of formulated breads is higher than 1. However, according to Scsg (2007), a good food menu should have a Ca/P ratio higher than 1. This is because foods high in phosphorus and low in calcium tend to make the body more acidic, deplete calcium and other minerals, and cause inflammation (Appiah et al., 2011). To avoid this problem, cinnamon‐enriched breads could help prevent the mineral imbalance between calcium and phosphorus in the body. Regarding phytochemical analysis, the formulated breads showed high polyphenol contents of 551.295 ± 25, 450.868 ± 64.13, and 292.345 ± 10.41 μg EAG/g DM, respectively, for the 1% bread, 0.5% bread, and the control bread. The phytochemical content increased with the incorporation rate of cinnamon powder in the breads. In addition, the Pearson correlation matrix showed a strong positive correlation between total phenols and antioxidant activity with a correlation coefficient *r* = 0.912 (*p* < .05). This correlation indicates that the presence of polyphenols in these samples would contribute significantly to their antioxidant properties. These results are in agreement with the work of Habellah et al. (2016) who demonstrated that the antioxidant properties of a plant extract are strongly related to its phytochemical composition and total phytophenolic content. The analyzed samples are rich in tannins and polyphenols that enter in the protection against cardiovascular and metabolic diseases (Traber et al., 2019). For Shori and Baba (2011), the incorporation of cinnamon in some foods significantly increases their phenolic content and antioxidant activity. Samples analyzed revealed that breads containing cinnamon each had high antioxidant activity compared to bread without cinnamon. Indeed, the goal of phenolic and antioxidant enrichment of foods is to improve the functionality of the food in preventing various diseases associated with oxidative stress (Bourgou et al., 2016). Cinnamon‐enriched breads could, therefore, help combat oxidative stress in the cell, leading to DNA, protein, and lipid damage and inducing many chronic diseases such as cancer, coronary heart disease, diabetes, neuronal disorders, mood disorders, and atherosclerosis, as well as other degenerative diseases and aging. The objective of the hedonic test was to know the organoleptic qualities of the breads enriched, respectively, with 0.5% and 1% of cinnamon powder by the various tasters through their opinions expressed by answering a series of questionnaires which were submitted to them during the tasting panel. At the end of the hedonic test, the results obtained thanks to the radar (or spider) diagrams revealed that the breads formulated with 0.5% cinnamon powder were globally appreciated by the panelists with the best surveys from the organoleptic quality point of view (smell, color, and taste) compared to the breads formulated with 1% cinnamon powder. The appreciation of these breads formulated with 0.5% cinnamon powder by the tasters would be due to the fact that these breads have organoleptic qualities close to those of the control bread (100% wheat). These results are consistent with those of Houede (2021) who worked on the production and quality control of bread by substituting wheat flour with pigeon pea flour in Djougou, northern Benin.

## 5 CONCLUSION

This study has demonstrated the influence of the incorporation of 0.5% and 1% of cinnamon powder in bread. It proved that cinnamon can be added to bread formulations as an ingredient capable of exerting a significant effect on the physicochemical, biochemical, phytochemical, functional, and nutritional composition of normal bread. The final goal is to contribute to the improvement of the nutritional and functional properties of bread. Phytochemical analysis revealed an increase in total phenols (551.295 ± 25 μg EAG/g DM), total flavonoids (53.117 ± 1.36 μg EQ/g DM), and condensed tannins (269.837 ± 39.25 μg EC/g DM) of the normal bread stick enriched with 1% cinnamon. This bread also presented the highest contents of protein (11.96 ± 0.02 g/kg) and crude fiber (1.70 ± 0.014 g/kg) with an energy value of 337.10 ± 0.74 kcal/100 g DM. From the organoleptic point of view, the bread with 0.5% cinnamon was more appreciated by the panelists than the bread with 1% cinnamon with an acceptability rate of 77% against 23% for the bread with 1% cinnamon. Based on these results, it appears that the bread with 1% cinnamon contributes best to the improvement of the functional and nutritional properties of white bread. Cinnamon has, therefore, a good nutritional potential to be used in the food industry and to develop new products. These new products could be used in the prevention of metabolic diseases.

## AUTHOR CONTRIBUTIONS

**Koffi Maïzan Jean‐Paul Bouatenin:** Conceptualization (equal); methodology (equal); writing – original draft (equal); writing – review and editing (equal). **Fatoumata Camara:** Supervision (equal). **Yabo Majoie Geroxie Tohoyessou:** Formal analysis (equal); methodology (equal). **Wahauwouélé Hermann Coulibaly:** Methodology (equal); visualization (equal). **Zamblé Bi Irié Abel Boli:** Visualization (equal). **Gniré Abibata Ouattara:** Methodology (equal). **Marina Koussemon:** Supervision (equal); visualization (equal).

## FUNDING INFORMATION

The author(s) received no financial support for the research, authorship, and/or publication of this article.

## CONFLICT OF INTEREST STATEMENT

The authors have no conflict of interest regarding the publication of the paper.

## CONSENT FOR PUBLICATION

All listed authors have read the final manuscript and provided consent for publication.

## ETHICS STATEMENT

This study does not involve any human or animal testing.

## Data Availability

The data files associated with this study have been submitted along with this manuscript and are available upon request. Please contact the corresponding author with any questions or concerns.
